# Preclinical evidence of remote ischemic conditioning in ischemic stroke, a metanalysis update

**DOI:** 10.1038/s41598-021-03003-6

**Published:** 2021-12-09

**Authors:** Coral Torres-Querol, Manuel Quintana-Luque, Gloria Arque, Francisco Purroy

**Affiliations:** 1 Clinical Neurosciences Group, Institut de Recerca Biomèdica de Lleida (IRBLleida), Lleida, Spain; 2 Epilepsy Unit, Neurology Department, Vall d’Hebron University Hospital, Universitat Autònoma de Barcelona, Barcelona, Spain; 3 Experimental Medicine Department, Universitat de Lleida, Lleida, Spain; 4 Medicine Department, Universitat de Lleida, Institut de Recerca Biomèdica de Lleida (IRBLleida), Lleida, Spain; 5 Stroke Unit, Department of Neurology, Universitat de Lleida, Hospital Universitari Arnau de Vilanova, Clinical Neurosciences Group IRBLleida, Avda Rovira Roure 80, 25198 Lleida, Spain

**Keywords:** Neuroscience, Cellular neuroscience, Molecular neuroscience, Neuro-vascular interactions

## Abstract

Remote ischemic conditioning (RIC) is a promising therapeutic approach for ischemic stroke patients. It has been proven that RIC reduces infarct size and improves functional outcomes. RIC can be applied either before ischemia (pre-conditioning; RIPreC), during ischemia (per-conditioning; RIPerC) or after ischemia (post-conditioning; RIPostC). Our aim was to systematically determine the efficacy of RIC in reducing infarct volumes and define the cellular pathways involved in preclinical animal models of ischemic stroke. A systematic search in three databases yielded 50 peer-review articles. Data were analyzed using random effects models and results expressed as percentage of reduction in infarct size (95% CI). A meta-regression was also performed to evaluate the effects of covariates on the pooled effect-size. 95.3% of analyzed experiments were carried out in rodents. Thirty-nine out of the 64 experiments studied RIPostC (61%), sixteen examined RIPreC (25%) and nine tested RIPerC (14%). In all studies, RIC was shown to reduce infarct volume (− 38.36%; CI − 42.09 to − 34.62%) when compared to controls. There was a significant interaction caused by species. Short cycles in mice significantly reduces infarct volume while in rats the opposite occurs. RIPreC was shown to be the most effective strategy in mice. The present meta-analysis suggests that RIC is more efficient in transient ischemia, using a smaller number of RIC cycles, applying larger length of limb occlusion, and employing barbiturates anesthetics. There is a preclinical evidence for RIC, it is safe and effective. However, the exact cellular pathways and underlying mechanisms are still not fully determined, and its definition will be crucial for the understanding of RIC mechanism of action.

## Introduction

Acute ischemic stroke (AIS) is the world’s second leading cause of mortality and the major cause of disability in adults worldwide^1^. The main revascularization therapies for AIS are thrombolysis with recombinant tissue-plasminogen activator (tPA) and endovascular thrombectomy. Unfortunately, many patients cannot benefit from those therapies due to mainly narrow therapeutic window and they can also induce ischemia–reperfusion injury (IRI)^2,3^. The development of novel therapeutic strategies is needed to extend therapeutic windows and to mitigate further brain injury.

Neuroprotective therapies have a great potential to not only increase the benefits of available reperfusion therapies but also to provide an advisable medical procedure for AIS patients who are not eligible for current treatments^4^. But translation of strategies targeting neuroprotection to the clinical practice has failed so far, despite extensive clinical trials^5^. In this scenario, a promising therapeutic approach, but insufficient traveled avenue, is the remote ischemic conditioning (RIC)^6^. Murry et al. first introduced the ischemic preconditioning therapy in a canine myocardial infarction model in 1986^7^. Since then, it has been repeatedly confirmed in animal models that ischemic preconditioning is a powerful endogenous protective strategy against IRI of multiple organs, including heart, brain and kidneys^8^. A significant breakthrough was the discovery that ischemic conditioning induction to a remote organ from the site of severe ischemia can also protect target tissue^9^. RIC consists of brief episodes of ischemia/reperfusion (I/R) in a distant organ, such as a limb, that can provide protection to the ischemic brain. It can be applied either before ischemia (pre-conditioning; RIPreC), during ischemia (per-conditioning; RIPerC) or after ischemia (post-conditioning; RIPostC) in a very simple way by using a blood pressure cuff on an arm. RIC is a safe, inexpensive, feasible, well tolerated, simple and harmless therapy for stroke; so, it has practical value^10^. The protective effect of RIC may be mediated by cellular mechanisms that counteract numerous aspects of stroke pathogenesis^11^. However, the specific underlying mechanisms contributing to RIC are complex and remain poorly understood^12^.

The interest of RIC in AIS has emerged in the last years. Three clinical trials have evaluated different strategies of RIC among AIS with mixed results^13–15^. In parallel, several clinical trials are now ongoing to investigate the efficacy of RIC in patients with acute stroke^16^. This systematic review and meta-analysis investigated basic preclinical studies of RIC in animal models of cerebral ischemia. Our aim was to elucidate the overall effects and variability of RIC on infarct volume in preclinical animal models compared with control group (no RIC application). Mainly, there are a number of unanswered questions: type of RIC application, number of limbs where RIC should be applied, number and length of RIC cycles of limb I/R and anesthetic used prior ischemia. We therefore identify the most suitable animal model to study the phenomenon of RIC and propose combinational scenarios with drugs that can amplify the beneficial effect of RIC. Finally, an updated of cellular pathways involved on RIC’s types was also performed.

## Material and methods

The systematic review protocol was registered on PROSPERO (CRD42020221321). The review protocol was prepared according to the preferred reporting items for systematic review and meta-analysis protocols statements (PRISMA-P)^17^. The systematic review report was written following the Preferred Reporting Items for Systematic review and Meta-Analysis (PRISMA) guidelines and the PRISMA checklist^18^. The search was first carried out in March 2020 and was repeated for an update by the first author in March 2021, in PubMed, SCOPUS and Web of Science (WOS) databases.

The search terms, strategy, and selection criteria are based on the PICOS system^19^ and were adapted to each database. PICOS-Parameters inclusion criteria were based on Population (animal models of ischemic stroke), Intervention (RIC: pre, per, post), Compare (RIC protocols/control/sham), Outcome (infarct volume), and Study design (experimental groups).

Articles were obtained by concatenating terms with boolean operators as follows: (“remote ischemic conditioning” OR “RIC” OR “limb ischemic conditioning” OR “Remote ischemic postconditioning” OR “remote postconditioning” OR “remote ischemic perconditioning” OR “remote ischemic preconditioning”) AND (“animal model” OR “mice” OR “rat”) AND (“stroke” OR “ischemic stroke” OR “ischaemia” OR “cerebral ischemia”). Studies were included from 2010 until present.

All studies were considered eligible if they investigated the effect of limb RIC (pre, per, or post) on cerebral ischemia animal models. No restrictions on species were applied. Studies were excluded if they did not investigate cerebral ischemia or did not apply RIC to a limb. Furthermore, studies were excluded if they specifically investigated hemorrhagic stroke model, if they applied RIC in humans and if the animal models had co-morbidities or risk factors. Reports were excluded if they were not available in English nor published in a peer-reviewed journals. Abstract articles, review articles, letters, proceedings paper or book chapters were also excluded.

One author (CT-Q) screened the title and abstract of each paper. After the screening, full texts were evaluated. For each study the following information when available was extracted: intervention, animal, gender, age, weight, animal model of ischemia, duration of ischemia, anesthetic used prior to ischemia, anesthetic used during RIC, RIC protocol, when RIC was started, RIC organ, outcomes reported, main pathway investigated and reference. For any missing or unclear data, the corresponding authors were contacted by e-mail to obtain the missing details.

CT-Q extracted the data from the selected studies. Data were manually entered into a Microsoft Excel spreadsheet (Version 14.0, 2010, Microsoft Corp., Redmond, California, USA); then reviewed, discussed and adjusted in accordance with the two reviewers (GA, FP). If needed, a consensus meeting and discussion resolved disagreement.

### Data analysis

All outcomes were transformed into effect sizes by using the studies’ reported statistics, mean and standard deviation or standard error, or results from analyses including t-tests, analysis of variance, correlations, regressions, and linear mixed-effects models.

The primary outcome was defined as the percentage of volume infarct reduction between RIC and control groups. The meta-analysis was conducted using the packages ‘tidyverse’, ‘meta’, ‘metafor’ and ‘dmetar’ of the R 4.0 software^20^. Studies presenting mean infarct size with standard deviation (SD) or standard error of mean (SEM) values in both intervention and control groups, were extracted for the meta-analysis. The effect size included was the difference in the mean percentage change (control–intervention), presented as the mean percentage change (95% confidence interval) in the infarct size of intervention group with respect to controls. As the SEM of the difference in percentage change was not reported, we first calculate the SD for each group (SEM*√n) to obtain the SEM of the difference (√[(SD12/n1) + (SD22/n2)]). Since the characteristics and methods of the interventions used in the studies are different, a random-effects model with the inverse variance method was performed to calculate the mean effect size. Forest plots were performed to show individual and global effect sizes.

Statistical heterogeneity across studies was evaluated using the Cochran's Q test and I2 statistic. I2 estimates the percentage of variation between all studies that is due to heterogeneity rather than chance; I2 > 50% is considered as substantial heterogeneity. The function *find.outliers* of the 'dmetar' package was used to explore for possible outliers and the function *InfluenceAnalysis* was used to detect studies with a high influence on the overall results.

A Baujat Plot was performed to plot the overall heterogeneity contribution and the influence on pooled results for each study in the meta-analysis. As heterogeneity was highly presented in the study and we performed meta-regressions and subgroup analyses to explore the effects of the different characteristics on the percentage change in infarct volume. Q statistic was used to assess difference in the subgroup analysis and random-effects linear regression models were performed to assess correlations with quantitative variables. The *bubble* function of the package 'meta' was used to plot meta-regression outputs.

Some quantitative variables (duration of cerebral ischemia, number of cycles and cycle duration) were divided into groups to have a different approach in a subgroup analysis. The analysis was also stratified by animal species, running separate meta-analyses for checking if the effects of some characteristics on the infarct size were different according to the animal tested.

### Ethical statement

This article does not contain any studies with human participants or animals performed by any of the authors.

## Results

**Figure 1:**
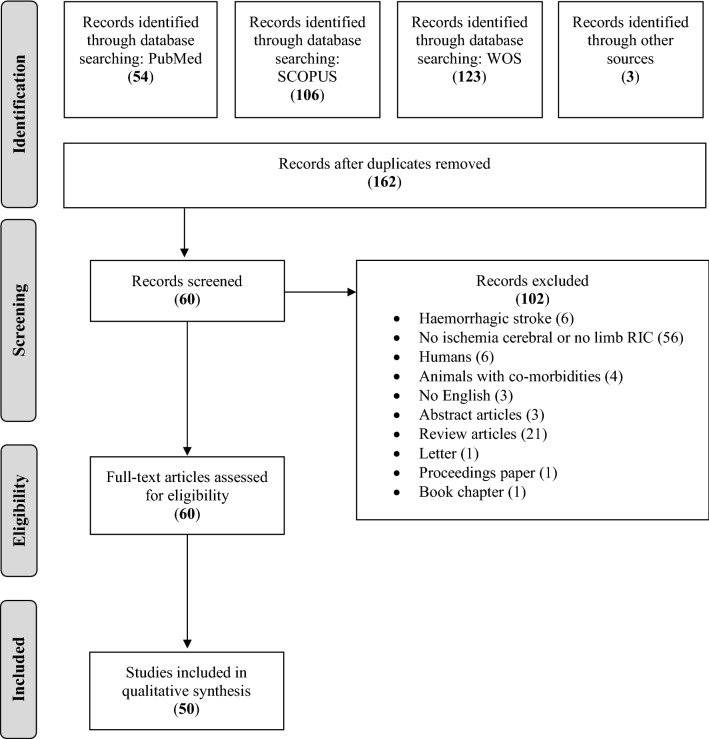
PRISMA flowchart of systematic review search process and meta-analysis on animal models of ischemic stroke and remote ischemic conditioning (RIC).

The results of the retrieved literature and selection process are presented in Fig. 1. The initial search identified 286 studies, of which 124 duplicates were removed, leaving 162 studies. After screening by title and abstract, 102 articles were rejected when exclusion criteria were applied: hemorrhagic stroke, articles not related to cerebral ischemia or limb RIC, articles related to humans, animal models with comorbidities, articles not available in English, review articles, letters, proceedings paper and a book chapter. The full text of the remaining 60 articles were read and 10 studies were excluded because there was no available data. Finally, fifty studies were included in the meta-analysis with data on 64 experiments.

The key characteristics of the included studies are presented in Table 1. Sixty-one of the 64 (95.3%) experiments we carried out in rodents: 52 used rats (81.2%) and nine studied mice (14.1%). Primates were only used in three experiments (4.7%). Almost all studies were performed in young and healthy animals. The majority of the studies used solely male (93.7%) animals and 6.3% of studies used solely female animals. The majority of publications induced transient focal cerebral ischemia (87.5%) with 20–120 min of middle cerebral artery occlusion (MCAo). Most studies included in this meta-analysis induced cerebral ischemia by intraluminal filament (84.3%) and seven studies used permanent models of middle cerebral artery (10.9%).

**Table 1:** Summarized description of selected studies characteristics on preclinical studies of RIPreC, RIPerC and RIPostC.

Intervention	Species	Gender, age, body weight	Animal model of ischemia	Duration of ischemia	Anesthetic used prior to ischemia	Anesthetic used during RIC	RIC protocol	When RIC was started	RIC organ	Reported outcomes	Molecular pathway investigated	Refs.
RIPreC	Swiss albino mice	Male, 20–25 g	2-VO model	Transient: 20 min	Thiopental sodium (45 mg/kg i.p)	Bosentan/propargylglycine/amino-oxyacetic acid	4 cycles 5′ × 5′	Brain ischemia was induced immediately after RIPreC	Left hind limb	↑ neurological function ↓ infarct size	Endothelin-1-CBS-CLS-H_2_S-Nrf2- Oxidative stress	^24^
	C57bl/6 mice	Male, 20–22 g	Electrocoagulation	Permanent	Not mentioned	Not mentioned	3 cycles 10′ × 10′	Not mentioned	Bilateral hind limbs	↑ neurological function ↓ infarct size	RIPC-induced exosomes contain increased levels of HIF-1α	^25^
	Sprague-Dawley rats	Male, 280–320 g	Intraluminal filament	Transient: 120 min	Isoflurane (4% induction and 2% maintenance)	2% Isoflurane	3 cycles, 5′ × 5′	Brain ischemia was induced at 1 h after RIPreC	Right hind limb	↑ neurological function ↓ infarct size	Activation of adenosine A1 receptor	^26^
	Sprague-Dwaley rats	Male, 280–320 g	Intraluminal filament	Transient. 90 min	Isoflurane (3–5% induction and 1–3% maintenance)	Isoflurane (1–3%)	3 cycles 10′ × 10′	Brain ischemia was induced 24 h after RIPreC	Bilateral hind limbs	↑ neurological function ↓ infarct size	HIF-1 α activation is a key factor of RIPC by mediating inflammation	^77^
	Sprague-Dawley rats	Male, 280–320 g	Intraluminal filament	Transient: 90 min	Isoflurane (5% induction and 2% maintenance)	Pentobarbital sodium salt (50 mg/kg) i.p	4 cycles 5′ × 5′	Brain ischemia was induced 1 h after RIPreC	Bilateral hind limbs	↑ neurological function ↓ infarct size	Increase B-cell population, increase m onocyte population, increase IL-6, increase TNFα (immune response)	^59^
	Sprague-Dawley rats	Male, adult (8–10 months), 250–300 g	Intraluminal filament	Transient: 120 min	Enflurane (4% induction and 2% maintenance)	Not mentioned	3 cycles 10′ × 10′ per day for 3 days	Brain ischemia was induced immediately after RIPreC	Bilateral hind limbs	↑ neurological function ↓ infarct size	No specific pathway mentioned	^68^
	Sprague-Dawley rats	Male, adult, 250–280 g	Intraluminal filament	Transient: 60 min	Chloral hydrate (10% 350 mg/kg i.p)	Not mentioned	4 cycles 5′ × 5′ for 3 days	Brain ischemia was induced after RIPreC	Left hind limb	↑ neurological function ↓ infarct size	RIPC activates the Notch1 and NF-KB pathways in neurons	^30^
	Sprague-Dawley rats	Male, 240–250 g	Intraluminal filament	Transient: 90 min	Chloral hydrate (400 mg/kg i.p)	Chloral hydrate (400 mg/kg i.p)	3 cycles 5′ × 5′	Brain ischemia was induced after RIPreC	Left hind limb	= neurological function ↓ infarct size	RIPC increases microparticles	^33^
	Sprague-Dawley rats	Male, P60	Intraluminal filament	Transient: 120 min	Isoflurane	Isoflurane	4 cycles 5′ × 5′	RPreC: 40 min before surgery RIPerC: 40 min before reperfusion	Left hind limb	N/R neurological function ↓ infarct size	Not mentioned	^78^
	Sprague-Dawley rats	Male, adult, 180–200 g	Intraluminal filament	Transient: 90 min	Not mentioned	Sodium pentorbital (30 mg/kg i.p)	3 cycles 10′ × 10′ up to 14 days before MCAO	Brain ischemia was induced after RIPreC	Upper tight	↑ neurological function ↓ infarct size	Not mentioned	^28^
	Sprague-Dawley rats	Male, 19–20 months, > 450 g	Intraluminal filament	Transient: 90 min	Isoflurane (3–5% induction and 1–3% maintenance)	1–3% isoflurane	3 cycles 10′ × 10′	Brain ischemia was induced 24 h after RIPreC	Both hind limbs	↑ neurological function ↓ infarct size	RIPC modulates the expression of HIF-1α and HIF-2α and reduces the expression of pro-inflammatory cytokines	^29^
	Sprague-Dawley rats	Male, 7–8-week-old, 260 g-280 g	Intraluminal filament	Transient: 120 min	﻿1% pentobarbital sodium salt (90 mg/kg i.p)	﻿1% pentobarbital sodium salt (90 mg/kg i.p)	4 cycles 5′ × 5′	Brain ischemia was induced 24 h after RIPreC	Hind limbs	↑ neurological function ↓ infarct size	RIPC prevents pJAK2, reducing the expression of pSTAT3, apoptosis and inflammation response	^31^
	Sprague-Dawley rats	Male, adult, 250–330 g	Intraluminal filament	Transient: 60 min	Isoflurane (5% induction and 2–3% maintenance)	Isoflurane (5% induction and 2–3% maintenance)	3 cycles 15′ × 15′	Brain ischemia was induced immediately after RIPreC	Left femoral artery	↑ neurological function ↓ infarct size	RIPC down-regulates aquaporin-4	^71^
	Sprague-Dawley rats	Male, 280–320 g	Intraluminal filament	Transient: 90 min	Isoflurane (5% induction and 2% maintenance)	Isoflurane (5% induction and 2% maintenance)	4 cycles 5′ × 5′	1 h before MCAO	Bilateral hind limb	↑ neurological function ↓ infarct size	During RIC there’s an immunomodulatory effect of the spleen	^36^
	White rats	Male, 320–350 g	Intraluminal filament	Transient: 60 min	Chloral hydrate (300 mg/kg i.p)	Without anesthesia, chloral hydrate or zoletil	3 cycles 5′ × 5′	24 h prior to brain ischemia	Bilateral hind limbs	↑ neurological function ↓ infarct size	RIC, chloral hydrate and zoletil produce a significant neuroprotective effect, but when togheter, not enhance the degree of neuroprotection	^79^
RIPerC	Sprague-Dawley rats	Male, adult, 250–280 g	Intraluminal filament	Transient: 120 min	10% chloral hydrate (350 mg/kg i.p)	Not mentioned	4 cycles 5′ × 5′	At 40 min prior to reperfusion	Left hind limb	↑ neurological function ↓ infarct size	Inhibits autophagy to attenuate plasma HMGB1 and induce neuroprotection	^39^
	C57BL/6J mice, ovariectomized	Female, 20 ± 2 weeks old	Embolic	Not mentioned	Mild isofluorane	Not mentioned	4 cycles 10′ × 10′	At 2 h post-stroke	Limb	↑ neurological function ↓ infarct size	RIPerC when combined with late IV-tPA decreased both Hb-content as well as edema	^42^
	C57BL/6J mice	Male, 20 ± 1 weeks old	﻿Embolic	Permanent	Isoflurane (3.5% induction and 1.5–2% maintenance)	Not mentioned	5 cycles 5′ × 5′	At 2 h post-stroke	Left hind limb	↑ neurological function ↓ infarct size	PI3k-Akt pathway	^80^
	Sprague-Dawley rats	Male, 300–320 g	Intraluminal filament	Transient: 120 min	Isoflurane 1.75%	Isoflurane 1.75%	3 cycles 10′ × 10′	RIC at 30 min of ischemia or during reperfusion	Bilateral hind limb	N/R neurological function ↓ infarct size	RIC involves AKT/Bcl2 phosphorylation (autophagy)	^37^
	Sprague-Dawley rats	Male, 2–5 months	2-VO model	Permanent	Isoflurane (4–5% induction and 1.5% maintenance)	Isoflurane (4–5% induction and 1.5% maintenance)	3 cycles 15′ × 15′	RIC at 60 min of ischemia	Bilateral hind limbs	N/R neurological function ↓ infarct size	RIC augmented collateral flow into distal MCA segments	^44^
	Sprague-Dawley rats	Male, adult, 280–320 g	Intraluminal filament	Transient: 90 min	1.5–3.5% enflurane	1.5–3.5% enflurane	3 cycles 10′ × 10′	RIC immediately after ischemia onset	Bilateral hind limb	↑ neurological function ↓ infarct size	RIC inhibits MMP9-mediated occluding degradation, decreasing BBB disruption	^41^
	Sprague-Dawley rats	Male, 280–320 g	Intraluminal filament	Transient: 120 min	10% chloral hydrate (0.35 ml/100 g i.p)	10% chloral hydrate (0.35 ml/100 g i.p)	4 cycles 10′ × 10′	RIC after 10 min of ischemia	Bilateral hind limbs	↑ neurological function ↓ infarct size	RIC activates autophagy/lysosomal pathway	^38^
RIPostC	C57BL/6 mice	Male, 8–10 weeks old, 26–30 g	Intraluminal filament	Transient: 60 min	Isoflurane (4% induction and 1.5% maintenance) or halothane (3% induction and 1% maintenance)	Isoflurane or ketamine-xylazine	3 cycles 5′ × 5′ for 3 days	90 min post-stroke	Bilateral hind limbs	↑ neurological function ↓ infarct size	LRIP under ketamine-xylazine anesthesia had better neurological deficit outcomes after stroke	^69^
	C57BL/6 mice	Male, adult, 20–22 g	Intraluminal filament	Transient: 45 min	Isoflurane (3–5% induction and 2% maintenance	Isoflurane 1–3%	3 cycles 10′ × 10′	Immediately after reperfusion	Hind limn	↑ neurological function ↓ infarct size	RIPostC modulated peripheral and brain inflammation during the brain injury induced by MCAO	^81^
	Sprague-Dawley rats	Female, 15–16 weeks old, 250–280 g	Intraluminal filament	Transient: 60 min	Chloral hydrate (350 mg/kg) i.p	Not mentioned	3 cycles 10′ × 10′	Immediately after reperfusion	Bilateral hind limbs	↑ neurological function ↓ infarct size	RIPostC decreased overexpression of MMP-9 and suppressed degradation of claudin-5	^82^
	Sprague-Dawley rats	Male, 250–280 g	Intraluminal filament	Transient: 90 min	Chloral hydrate (330 mg/kg, i.p)	Not mentioned	3 cycles 5′ × 5′	0, 1 and 3 h after reperfusion	Left femoral artery	↑ neurological function ↓ infarct size	RIPostC inhibites the activation of NADPH oxidase in neutrophils	^52^
	Sprague-Dawley rats	Male, 250–280 g	Intraluminal vascular occlusion	Transient: 60 min	Chloral hydrate (1 ml/100 g, i.p)	Not mentioned	3 cycles 10′ × 10′	At the begginning of reperfusion	Proximal hind limbs	↑ neurological function ↓ infarct size	LRIP exhibits a protective effect through the suppression of HIF-1α	^58^
	Sprague-Dawley rats	Male, 300–320 g	Intraluminal vascular occlusion	Transient: 120 min	Isoflurane (1.75%)	Not mentioned	3 cycles 10′ × 10′	0 and 10 min of reperfusion	Bilateral femoral artery	↑ neurological function ↓ infarct size	AKT/GSK3b-dependent autophagy	^60^
	Sprague-Dawley rats	Male 280–320 g	Intraluminal filament	Transient: 120 min	Sodium pertobarbital 1% (40 mg/kg i.p)	Sodium pertobarbital 1% (40 mg/kg i.p)	3 cycles 10′ × 10′	10 min after reperfusion	Right femoral arteries	↑ neurological function ↓ infarct size	RPostC alleviated cerebral reperfu- sion injury through ROS-mediated inhibition of endoge- nous PKC activation signaling cascade	^51^
	Sprague-Dawley rats	Female, adult, 250–280 g	Intraluminal filament	Transient: 60 min	Chloral hydrate i.p	Not mentioned	3 cycles 10′ × 10′	Immediately after MCAO	Bilateral hind limbs	↑ neurological function ↓ infarct size	RIPostC inhibites the overexpression of TLR4 and NF-KB	^57^
	CD1 mice	Male, adult, 25–30 g	Intraluminal filament	Transient: 60 min	10% Chloral hydrate	Not mentioned	3 cycles 5′ × 5′	At the beginning of reperfusion	Bilateral hind limb	↑ neurological function ↓ infarct size	RIPostC reduces oxidative stress and activates the Nrf2-ARE pathway	^50^
	Sprague-Dawley rats	Male, adult, 290–330 g	Electrocoagulation	Transient: 30 min	Enflurane (2–4%)	Not mentioned	3 cycles 10′ × 10′	Immediately after stroke onset	Bilateral lower limbs	↑ neurological function ↓ infarct size	Bcl-2 is upregulated, while cleaved-caspase-3 is downregulated	^83^
	Sprague-Dawley rats	Male, 280–310 g	Intraluminal filament	Transient: 120 min	Isoflurane (1.75%)	Not mentioned	3 cycles 10′ × 10′	Immediately after ischemia	Bilateral hind limbs	↑ neurological function ↓ infarct size	RIPostC attenuates ER stress-dependent apoptotic signaling	^21^
	Sprague-Dawley rats	12-week old, 250–280 g	Intraluminal filament	Transient: 120 min	Chloral hydrate (350 mg/kg i.p)	Chloral hydrate (350 mg/kg i.p)	3 cycles 5′ × 5′	Beginning reperfusion	Ipsiateral hind limb	↑ neurological function ↓ infarct size	RIPostC downregulates RGMA, IL-1B and IL-6	^56^
	Sprague-Dawley rats	Male, 270–330 g	Intraluminal filament	Permanent occlusion of dMCA + 30 min occlusion bCCA	Isoflurane (5% induction and 1–2% maintenance)	Isoflurane	3 cycles 15′ × 15′	0 h, 3 h or 6 h after reperfusion	Left hind limb	↑ neurological function ↓ infarct size	RIPostC protects against ischemia via the nerve pathway and via modulating protein synthesis	^84^
	C57BL/6 J mice	Male, 9–10 weeks old	3-VO	Permanent occlusion of dMCA + 15 min occlusion dMCA	Enflurane (1–2.5%)	Not mentioned	3 cycles 10′ × 10′	Immediately after CCAs release	Bilateral lower limbs	↑ neurological function ↓ infarct size	RIPostC enhances leptomeningeal collateral circulation	^49^
	Rhesus monkeys	Male, 2.3 ± 0.42 years, 8.25 ± 0.65 kg	Thromboembolic clot	Permanent	Ketamine (10 mg/kg i.m) + Propofol (0.5 mg/kg per h)	Propofol (0.5 mg/kg per h)	10 cycles 5′ × 5′	Immediately after stroke	One, two or 4 limbs	↑ neurological function = infarct size	Two-limb RIPC reduced cardiac enzymes, vascular endothelial injury and inflammatory responses	^23^
	Sprague-Dawley rats	Male, 250–300 g	Intraluminal filament	Transient: 90 min	10% Chloral hydrate i.p	Not mentioned	3 cycles 10′ × 10′ during 21 days	2 days after MCAO	Hind limb	↑ neurological function ↓ infarct size	RIP up-regulates endogenous tissue kallikrein	^54^
	Sprague-Dawley rats	Male, adult, 280–320 g	Intraluminal filament	Transient: 90 min	Pentobarbital sodium (50 mg/kg i.p)	Pentobarbital sodium (50 mg/kg i.p)	3 cycles 10′ × 10′	At the beginning of cerebral reperfusion	Bilateral hind limbs	↑ neurological function ↓ infarct size	RIC increases mitochondrial autophagy and inhibites oxidative stress by up-regulating Parkin and DJ-1 protein expression	^53^
	Sprague-Dawley rats	Male, 10–week-old, 220–280 g	Intraluminal filament	Transient: 120 min	Enflurane (4% induction and 2% maintenance)	Not mentioned	3 cycles 10′ × 10′	8 and 24 h after reperfusion	Bilateral femoral arteries	= neurological function ↓ infarct size	RIC inhibites NF-KB expression and increases Bcl-2 expression	^76^
	Sprague-Dawley rats	Male, 330–380 g	Intraluminal filament	Transient: 120 min	10% chloral hydrate i.p	Not mentioned	3 cycles 15′ × 15′	At the same time as reperfusion	Left limb	= neurological function ↓ infarct size	RIC reverses the eNOS uncoupling induced by IRI	^45^
	Sprague-Dawley rats	Male, adult, 250–280 g	Intraluminal filament	Transient: 90 min	10% chloral hydrate (300 mg/kg i.p)	Not mentioned	1, 2 or 3 cycles for (5/5, 10/10 or 15/15)	At the beginning of reperfusion	Bilateral femoral arteries	↑ neurological function ↓ infarct size	RIC exhibits its maximum protective effect if limb occlusion/reperfusion lasts 40–60 min	^85^
	Sprague-Dawley rats	Male, 250–280 g	Intraluminal filament	Transient: 120 min	Chloral hydrate (350 mg/kg i.p)	Chloral hydrate (350 mg/kg i.p)	3 cycles 15′ × 15′	Just before MCA reperfusion	Right hind limb	↑ neurological function ↓ infarct size	RIC inhibites autophagy through the mTOR/p70S6K pathway	^86^
	Sprague-Dawley rats	Male, 260–280 g	Intraluminal filament	Transient: 90 min	10% Chloral hydrate (300 mg/kg i.p)	Not mentioned	3 cycles 10′ × 10′	Immediately after MCAO	Bilateral hind limbs	↑ neurological function ↓ infarct size	RIC induced neurogenesis both in the SGZ and SVZ	^87^
	Wistar rats	Male, 280–330 g	Intraluminal filament	Transient: 90 min	Isoflurane 1–2%	Isoflurane 1–2%	3 cycles 10′ × 10′	Immediately after reperfusion	Bilateral hind limbs	↑ neurological function ↓ infarct size	RIC inhibites apoptosis molecules of the mitochondrial pathway	^88^
	Sprague-Dawley rats	Male, 250–300 g	Intraluminal filament	Transient: 100 min	2% sevoflurane	2% sevoflurane	Several cycles and time intervals of I/R	Immediately after reperfusion	Femoral artery occlusion	↑ neurological function ↓ infarct size	RIC involves p-ERK and nNOS	^47^
	Sprague-Dawley rats	Male, 290–310 g	Intraluminal filament	Transient: 90 min	Pentobarbital sodium (50 mg/kg i.p)	0.25% Bupivacaine hydrochloride	2–3 cycles 15′: 3 cycles 5′	3 h or 6 h after reperfusion	Bilateral hind limbs	↑ neurological function ↓ infarct size	RIC inhibits apoptotic injury through opening K_ATP_ channels	^62^
	Sprague-Dawley rats	Male, 300–320 g	Intraluminal filament	Transient: 120 min	Chloral hydrate (350 mg/kg i.p)	Not mentioned	3 cycles 10′ × 10′	At the beginning of reperfusion	Bilateral hind limbs	↑ neurological function ↓ infarct size	RIC induced elevation of fibulin-5 and activation of the AKT pathway	^89^
	Sprague-Dawley rats	Male, 250–300 g	Intraluminal filament	Transient: 90 min	Chloral hydrate (300 mg/kg i.p)	Not mentioned	3 cycles 5′ × 5′	At the beginning of reperfusion	Right hind limb	= neurological function ↓ infarct size	RIC upregulates STAT3 and reduces apoptosis	^61^
	Sprague-Dawley rats	Female, 7 weeks, 250–280 g	Intraluminal filament	Transient: 60 min	10% Chloral hydrate i.p	Not mentioned	3 cycles 10′ × 10′	After MCAO	Bilateral hind limbs	↑ neurological function ↓ infarct size	RIC downregulates AQP4 in astrocytes	^48^

The most commonly RIC protocol employed was three to four repetitions of 5–15 min I/R using a pressure cuff, applied on one (37.5%) or two (60.9%) limbs to observe a neuroprotective effect. Thirty-nine of the 64 experiments studied RIPostC (61%), sixteen examined RIPreC (25%) and nine tested RIPerC (14%). In 58 studies, RIC was performed as a single application (90.6%) and six studies employed multiple applications (9.4%). The anesthetic used varied between studies, being chloral hydrate the most used (36%) (Table 1).

### Infarct volume’s dependent factors

**Figure 2:**
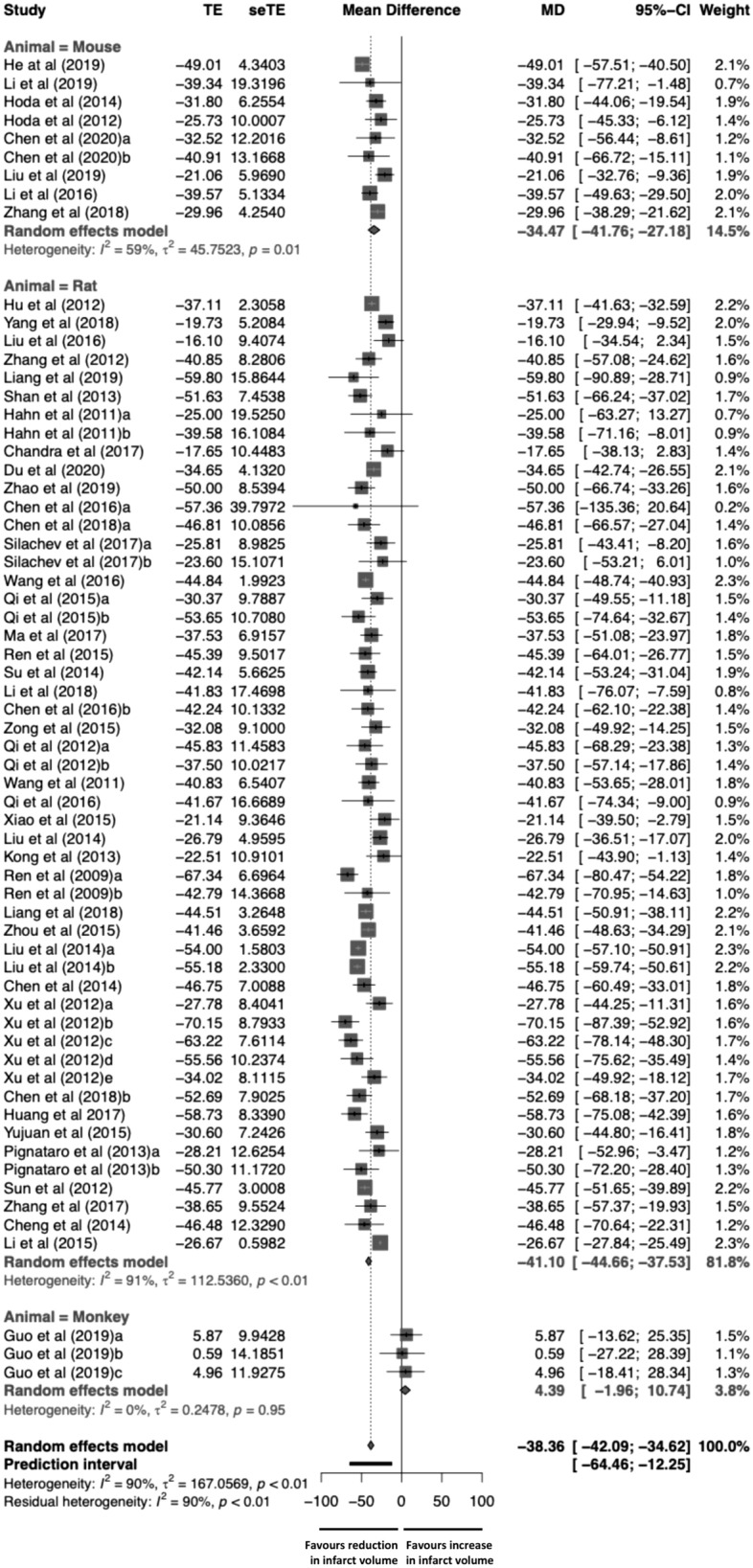
Forest plot to illustrate the efficacy of remote ischemic conditioning on infarct volume by animal model from 64 analyzed experiments. Forest plot of mean difference (MD) and their 95% CI for individual trials determined from the result of 64 trials comparing the effect of remote ischemic conditioning with control on infarct volume. Studies are grouped by species. The solid vertical line represents a mean difference of 0 or no effect. Points to the left of the line represent a reduction in infarct volume, and points to the right of the line indicate an increase. Each square around the point effect represents the mean effect size for that study and reflects the relative weighting of the study to the overall effect size estimate. The larger the box, the greater the study contribution to the overall estimate. The weight that each study contributed is in the right-hand column. *MD* mean difference, *CI* confidence interval.

**Figure 3:**
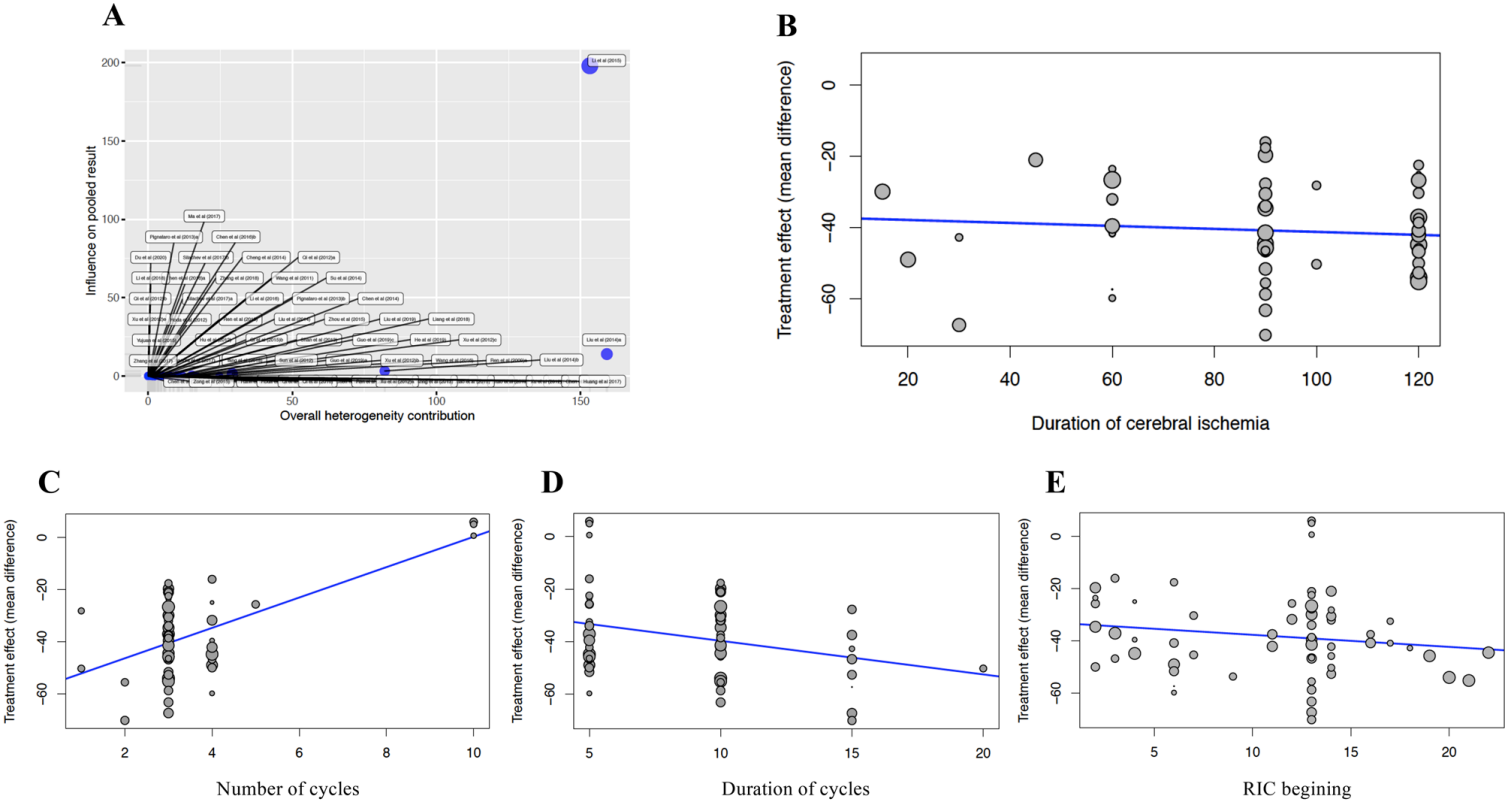
Impact of studied factors on infarct volume evaluated by meta-analysis comparisons of all included species. (**A**) The Baujat plot shown which studies contributed to greater heterogeneity^76^ and what were the most influential studies on the overall result^48^. (**B**) Duration meta-regression graph. There was not greater reduction in volume to longer duration of cerebral ischemia (p = 0.465). (**C**) Number of cycles meta-regression graph. There was less reduction in volume with a greater number of cycles (p < 0.001). (**D**) Duration of cycles (min) meta-regression graph. There was a greater reduction in volume as the duration of the cycles increases (p = 0.0165). (**E**) Conditioning start time meta-regression graph. There was no significant volume reduction based on conditioning onset time (p = 0.205).

The meta-analysis included data from 941 animals (779 [82.8%] rats, 138 [14.7%] mice, 24 [2.6%] monkeys), 468 (49.7%) animals were control and 473 (50.3%) animals that underwent RIC. A random effect model showed that RIC was significantly effective when compared to control group (− 38.36%; 95% CI − 42.09 to − 34.62%; 95% PI [prediction interval], − 64.46 to − 12.25%; p ≤ 0.0001) (Fig. 2). However, high heterogeneity between studies was detected (I^2^ = 90.1%; Q = 635.72, df = 63, *p* < 0.0001). The variance of the distribution of the effect sizes in this samples was T^2^ = 167.06 (Table 2). Figure 3A presented results of the influence analysis. The study that contributed to a higher heterogeneity^21^ and the most influential study on the overall results^22^ were identified (Fig. 3A).

**Table 2:** The effect of infarct volume on different analyzed factors in all species compared to controls.

	Infarct volume reduction by species (%)			
	Volume reduction (all) Median (95% CI)	Volume reduction (rats) Median (95% CI)	Volume reduction (mice) Median (95% CI)	Volume reduction (monkeys) Median (95% CI)
Pooled	− 38.4% (− 42.1%, − 34.6%)	− 41.1% (− 44.7%, − 37.5%)	− 34.5% (− 41.7%, − 27.2%)	+ 4.4% (− 1.9%, + 10.7%)
Prediction interval	(− 64.5%, − 12.3%)	(− 62.7%, − 19.5%)	(− 52.1%, − 16.8%)	(− 15.4%, + 24.2%)
Test of heterogeneity	I^2^ = 90.1%, **p < 0.001**	I^2^ = 24.7%, p = 0.076	I^2^ = 58.8%, **p = 0.013**	I^2^ = 0%, p = 0.953
**Analyzed factors**				
Intervention				
RIPreC	− 36.2% (− 43.4%, − 29.1%)	− 34.7% (− 42.6%, − 26.9%)	− 48.4% (− 77.4%, − 19.5%)	–
RIPerC	− 39.7% (− 45.7%, − 33.7%)	− 42.7% (− 47.8%, − 37.5%)	− 30.1% (− 65.0%, + 4.9%)	–
RIPostC	− 38.8% (− 44.3%, − 33.3%)	− 43.4% (− 48.1%, − 38.6%)	− 31.6% (− 41.5%, − 21.6%)	+ 4.4% (− 1.9%, + 10.7%)
p-value	p = 0.709	p = 0.112	**p < 0.001**	–
Ischemia model				
Transient ischemia	− 40.8% (− 44.2%, − 37.5%)	− 41.6% (− 45.2%, − 37.9%)	− 35.6% (− 46.6%, − 24.5%)	–
Permanent ischemia	− 15.9% (− 33.8%, + 1.9%)	− 30.6% (− 133.5%, + 72.4%)	− 28.8% (− 101.5%, + 43.8%)	+ 4.4% (− 1.9%, + 10.7%)
p-value	**p < 0.001**	p = 0.185	p = 0.347	–
Duration of ischemia (min)				
Coef (95% CI)	− 0.042 (− 0.156, + 0.072)	− 0.004 (− 0.160, + 0.152)	+ 0.233 (− 0.621, + 0.734)	–
p-value	p = 0.465	p = 0.958	p = 0.827	–
Duration of ischemia (grouped, min)				
< 90’	− 37.5% (− 44.8%, − 30.3%)	− 39.6% (− 51.3%, − 27.9%)	< 60: − 33.7% (− 69.1%, + 1.7%) 60: − 38.7% (− 46.5%, − 31.0%)	–
90’–100’	− 41.4% (− 48.0%, − 34.8%)	− 41.4% (− 48.0%, − 34.8%)	–	–
> 100’	− 43.0% (− 47.6%, − 38.4%)	− 43.0% (− 47.6%, − 38.4%)	–	–
p-value	p = 0.398	p = 0.799	p = 0.547	–
Number of RIC cycles				
Coef. (95%CI)	+ 5.817 (+ 3.571, + 8.064)	+ 2.511 (− 4.234, + 9.256)	− 1.876 (− 14.210, + 10.457)	–
p-value	**p < 0.001**	p = 0.458	p = 0.729	–
Number of RIC cycles (grouped, n)				
< 3	− 52.5% (− 79.7%, − 25.2%)	− 52.5% (− 79.7%, − 25.2%)	–	–
3	− 39.2% (− 42.8%, − 35.6%)	− 40.1% (− 44.1%, − 36.2%)	− 31.8% (− 40.1%, − 23.6%)	–
> 3	− 30.8% (− 43%, − 18.6%)	− 41.2% (− 51.7%, − 30.7%)	− 37.4% (− 67.6%, − 7.1%)	+ 4.4% (− 1.9%, + 10.7%)
p-value	p = 0.101	p = 0.373	p = 0.475	–
Length of each RIC cycle (min)				
Coef. (95% CI)	− 1.282 (− 2.321, − 0.242)	− 0.874 (− 1.893, + 0.146)	+ 2.422 (+ 0.030, + 4.815)	–
p-value	**p = 0.016**	p = 0.091	**p = 0.048**	–
Length of each RIC cycle (grouped, min)				
5’	− 33.9% (− 41.2%, − 26.7%)	− 39.3% (− 45.3%, − 33.3%)	− 40.2% (− 51.3%, − 29.1%)	+ 4.4% (− 1.9%, + 10.7%)
10’	− 38.3% (− 42.8%, − 33.8%)	− 39.7% (− 44.6%, − 34.8%)	− 28.3% (− 37.2%, − 19.4%)	–
≥ 15’	− 49.9% (− 61%, − 38.8%)	− 49.9% (− 61.0%, − 38.8%)	–	–
p-value	**p = 0.026**	p = 0.131	**p = 0.015**	–
Number of limbs where RIC was applied				
1	− 39.1% (− 45.7%, − 32.5%)	− 43.5% (− 49.2%, − 37.8%)	− 32.9% (− 53.3%, − 12.5%)	+ 5.9% (− 13.6%, + 25.3%)
2	− 38.8% (− 43.2%, − 34.5%)	− 39.9% (− 44.6%, − 35.3%)	− 34.6% (− 41.2%, − 28.0%)	+ 0.6% (− 27.2%, + 28.4%)
4	+ 5.0% (− 18.4%, + 28.3%)	–	–	+ 5.0% (− 18.4%, + 28.3%)
p-value	**p = 0.013**	p = 0.315	p = 0.810	p = 0.953
When RIC was started				
Before	− 37.2% (− 43.7%, − 30.5%)	− 36.0% (− 42.8%, − 29.1%)	− 49.0% (− 57.5%, − 40.5%)	–
During	− 37.8% (− 45.8%, − 29.8%)	− 41.2% (− 50.5%, − 32.0%)	− 30.0% (− 65%, + 4.9%)	–
After	− 38.8% (− 44.3%, − 33.3%)	− 43.4% (− 48.1%, − 38.6%)	− 31.6% (− 41.5%, − 21.6%)	+ 4.4% (− 1.9%, + 10.7%)
p-value	p = 0.917	p = 0.177	**p < 0.001**	–
When RIC was started (ca. ordinate)				
Coef. (95% CI)	− 0.461 (− 1.180, 0.258)	− 0.732 (− 1.331, − 0.133)	+ 1.842 (− 0.303, + 3.986)	–
p-value	p = 0.205	**p = 0.018**	p = 0.080	–
Anesthetic used prior ischemia				
Thiopental sodium	− 49% (− 57.5%, − 40.5%)	–	− 49% (− 57.5%, − 40.5%)	–
Isoflurane	− 34.8% (− 40.5%, − 29.0%)	− 36.7% (− 43.5%, − 29.9%)	− 26.8% (− 36.0%, − 17.6%)	–
Enflurane	− 42.5% (− 56.5%, − 28.4%)	− 45.4% (− 61.9%, − 28.9%)	− 29.9% (− 38.3%, − 21.6%)	–
Halothane	− 40.9% (− 66.7%, − 15.1%)	–	− 40.9% (− 66.7%, − 15.1%)	–
Chloral hydrate	− 43.4% (− 48.7%, − 38.0%)	− 43.6% (− 49.3%, − 38.0%)	− 39.6% (− 49.6%, − 29.5%)	–
Pentobarbital sodium	− 44.0% (− 49.1%, − 38.9%)	− 44.0% (− 49.1%, − 38.9%)	–	–
Zoletil	− 23.6% (− 53.2%, + 6.0%)	− 23.6% (− 53.2%, + 6.0%)	–	–
Sevoflurane	− 40.0% (− 180%, + 100%)	− 40.0% (− 180%, + 100%)	–	–
Ketamine + Propofol	+ 4.4% (− 1.9%, + 10.7%)	–	–	+ 4.4% (− 1.9%, + 10.7%)
p-value	**p < 0.001**	p = 0.297	**p < 0.001**	–
Sex				
Male	− 38.9% (− 42.9%, − 34.9%)	− 41.9% (− 45.6%, − 38.2%)	− 34.8% (− 43.3%, − 26.3%)	+ 4.4% (− 1.9%, + 10.7%)
Female	− 28.9% (− 36.6%, − 21.1%)	− 28.8% (− 44.8%, − 12.8%)	− 31.8% (− 44.1%, − 19.5%)	–
p-value	**p = 0.001**	**p = 0.002**	p = 0.675	–

Significant values are in [bold].

Subgroup analysis and meta-regression was performed using random-effects model. No significant differences were observed on the type of intervention when all species were analyzed: RIPreC (− 36.2%; 95% CI − 43.4 to − 29.1%), RIPerC (− 39.7%; 95% CI − 45.7 to − 33.7%) and RIPostC (− 38.8%; 95% CI − 44.3 to − 33.3%) (p = 0.709 between groups). In mice, the major effect was significantly observed in RIPreC (− 48.4%; 95% CI − 77.4 to − 19.5%; p < 0.001). In contrast to studies performed on rats and mice, in the three studies performed on monkeys RIPostC showed a tendency to increase the infarct volume (+ 4.4%; 95% CI − 1.96%, + 10.74%, p = 0.097). The reduction in infarct size was significantly higher in transient ischemia studies (− 40.8%; 95% CI − 44.2 to − 37.5%) than in permanent ischemia studies (− 16%; 95% CI − 33.8 to + 1.9%) (p < 0.001). However, the duration of ischemia did not show a time-dependent effect (− 0.042%; 95% CI − 0.156 to + 0.072%; p = 0.465) (Fig. 3B).

Infarct size was significantly increased when a higher number of RIC cycles were applied (+ 5.817; 95% CI + 3.571 to + 8.064%; p < 0.001) (Fig. 3C). When all species (mice, rats, monkeys) were considered, volume was significantly reduced when the cycle duration increased (− 1.282%; 95% CI − 2.321 to − 0.242%; p = 0.016) (Fig. 3D). However, in mice the observation was the opposite: studies that applied 10-min RIC cycles described higher volume reduction than studies that used 5-min RIC cycles.

When RIC was applied to one limb (− 39.1%; 95% CI − 45.7 to − 32.5%) the effect was similar to when it was applied to two limbs (− 38.8%; 95% CI − 43.2 to − 34.5%). Only in one study, which used monkeys, RIC was applied to four extremities (+ 5.0%; 95% CI − 18.4 to + 28.3%)^23^. The results of this study within the meta-analysis showed significant differences (p = 0.013) regarding the number of limbs. However, if this study was not included in the meta-analysis, differences were not observed on the variable number of limbs (p = 0.604). Initiation of RIC was not related with the infarct volume reduction (− 0.461%; 95% CI − 1.180 to − 0.258%; p = 0.205) (Fig. 3E). Finally, significant sex-differences were observed in experiments performed on rats but not on mice. Experiments performed on male animals obtained higher proportion of volume reduction than experiments performed on female animals (− 41.9%; 95% CI − 45.6 to − 38.2% vs. − 28.8%; 95% CI − 44.8 to − 12.8%; p = 0.002), which it might indicate a different RIC’s mechanism of action by sex.

### Effect of anesthetic on infarct volume reduction

Up to nine different anesthetic strategies were used in the experiments. Among them, chloral hydrate was used in 22 experiments and isoflurane was used in 20, both were the most represented. The combination of ketamine and propofol was only used in monkey experiments. Significant differences were observed in experiments performed on mice (p < 0.001), due to anesthetic strategy. In addition, when we compared the two most frequent used types of anesthesia, chloral hydrate (− 43.37%; 95% CI − 48.73 to − 38.00%) showed a greater infarct volume reduction than isoflurane (− 34.76%; 95% CI − 40.48 to − 29.05%, (p = 0.022).

### Pathophysiology of RIC effects

**Figure 4:**
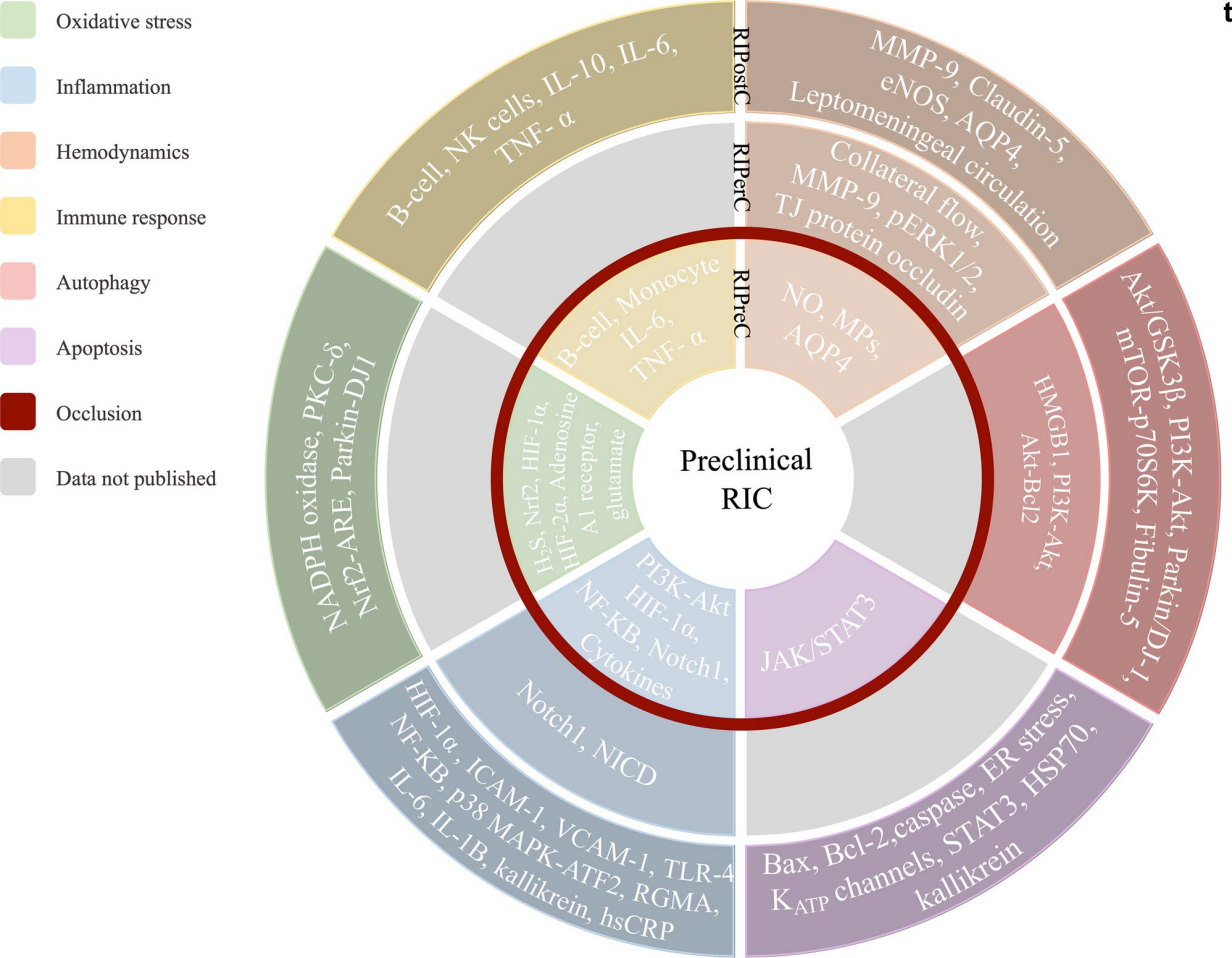
Summary of the proposed cellular mechanisms involved on remote ischemic conditioning (RIC).

Figure 4 illustrates the schematic representation of suggested underlying mechanisms of RIPreC, RIPerC and RIPostC. Selected studies had also described molecular and cellular processes involved on RIC. Diagram showed the different mechanisms grouped by cellular processes related with ischemic damage: oxidative stress, inflammation, hemodynamics, immune response, autophagy, and apoptosis. However, many molecular pathways were described, none was translated to humans. Special consideration should be given to four spots where no data was reported: no autophagic pathway was related to RIPreC and RIPerC molecular underlying mechanisms were not described on apoptosis, oxidative stress, and immune response.

RIPreC would decrease oxidative stress through the release of endothelin-1 and the increase of H_2_S, Nrf2, HIF-1α, SOD1 and HO1^24–28^. It would also reduce neuroinflammation by modulating the expression of HIF-1α, HIF-2α and activating the Notch1 and NF-KB pathways^25,29,30^. Apoptosis has been shown to be reduced when preconditioning is applied by regulating the JAK2/STAT3 signalling pathway^31^. Also, an improvement of brain edema and downregulation of the expression of AQP4 is observed^32,33^. Several studies have shown that RIPreC modulates the immune response decreasing the levels of IL-10, IL-6 and TNFα in the blood^34–36^. RIPerC would inhibit the autophagy process by increasing Bcl-2 phosphorylation^37–39^, decrease inflammation through incrementing Notch and NICD expressions^40^ and increase collateral circulation^41–44^. Finally, RIPostC would decrease brain edema and blood–brain barrier permeability via upregulating eNOS, decreasing MMP-9 and increasing claudin-5 expression^41,45–49^. Multiple preclinical studies have shown that RIPostC could reduce oxidative stress through upregulation of Nrf2 along with HO1, NQO1 and Parkin/Dj-1^50–53^. RIPostC has been shown to protect against ischemic injury by downregulating proinflammatory pathways^22,23,54–58^ and improving the peripheral immune response^36,59^. Diverse mechanisms have been proposed for RIPostC-mediated autophagy, including increase of AKT/GSK3β-dependent activation, induction the mitophagy via up-regulation of Parkin/DJ-1 proteins expression and activation of the mTOR/p70S6K signaling pathway^53,60^. Other studies demonstrated that RIPostC treatment upregulate Bcl-2 and heat-shock protein 70 (HSP70) expression and downregulate Bax expression, attenuating apoptosis^21,46,54,61–63^.

## Discussion

This systematic review and meta-analysis summarized the evidence on the protective effects of RIC on infarct volume in preclinical stroke models. A total of fifty studies with data on 64 experiments were included, which involved 941 animals. In all studies, the reduction in infarct volume in RIC groups compared to control was 38.4%. Our results suggested that RIC is more efficacious in transient than permanent ischemia, applying a smaller number of RIC cycles, using a RIC cycle length of ≥ 15 min, using one or two limbs, employing barbiturates anesthetics and in male animals.

The majority of papers in this review used rodents, predominately rats. Despite being the most applicable animal models for research related to stroke, the demand for larger models, such as rabbits and even nonhuman primates, is increasing to better understand the disease and RIC mechanism of action^64^.

Most RIC studies used transient focal cerebral ischemia with intraluminal suture stroke model because it closely mimics the human ischemic stroke^65^. The optimal conditioning protocol for RIC to elicit organ protection remains unknown. Less than three cycles or more than 15 min of treatment intensity can have a significant role in ischemic neuroprotection. However, more than three ischemic cycles or cycles < 5 min did not have such a neuroprotective effect. The present evidence suggests that there may be a minimum threshold value for the neuroprotective effect of RIC. RIC was beneficial in all three temporal variants after its initial application: RIPreC, RIPerC and RIPostC. Despite this, RIC was found most effective when delivered after stroke injury (RIPostC) followed by the application during stroke (RIPerC). Both approaches are suitable to be translated to patients, where RIC would be applied during ambulance transportation once admission at the emergency room is done or during the first 24 h after the stroke. The preclinical evidence supported the current clinical trials on-going on RIPerC and RIPostC. Interestingly, the reduce in infarct size is related to neurological functional improvement.

Most studies performed RIC as a single application. A single bout of RIC activates at least 2 distinct time frames of neuroprotection against I/R injury of the brain. The initial neuroprotection is short-lasting (2 h) and occurs immediately after RIC^66^. The delayed form of neuroprotection reappears after 12–24 h and lasts 48–72 h^67^. In addition to the short-lasting benefits of a single bout of RIC, long-term benefits may be induced with repeated daily conditioning^54^. A limited number of studies have explored the effect of repeated RIC in an animal model for brain ischemia^28,30,68,69^. RIC reduced infarct volume in both male and female animals but provided significantly more protection in males. It must be pointed out that only four studies examined the effect of RIC in female animals, so more experimental research on female animals should be done to determine the RIC effects on female animals.

Both rat and mice studies demonstrated significant statistical reduction in infarct volume in RIC groups compared to controls. Subgroup analysis shown that in mice experiments, there was a significant interaction with RIPerC. Subspecies analysis showed no significant interaction with duration of ischemia and number of RIC cycles. However, our analysis demonstrated > 100 min of ischemia to be more effective than < 90 min in rats. Similarly, 60 min of ischemia was more powerful than < 60 min in mice. We found 3 and > 3 cycles to be equally effective in rats, being < 3 the most beneficial. Conversely, > 3 cycles in mice provided a greater neuroprotection. These differences might be related with the total ischaemic dose (cycle number and duration). Interestingly, in rats, doses above 15 min were more effective, while in mice the opposite occurs. The shorter the length of each RIC cycle, the better reduction of the infarct size.

In all species, significant sex-differences were observed in experiments performed on rats but not on mice, showing a significant effect on males. This observation would be explained by the interaction of female’s hormones with the RIC’s molecular cascade and that most of the studies were performed in male mice. Taking in consideration the sex differences is particularly important because of the translational goal, and it could lead to better treatments for cerebrovascular diseases if RIC might have a differential sex-effect.

Our analysis supports the previous findings of no significant differences in RIC effect when it was applied on one or two limbs^70^. We also noticed a reduction of efficacy if isoflurane is used during surgical procedure^36,71^. Signaling protective pathways associated with the induction of brain ischemic tolerance are known for the inhalational anesthetics, however very little is known about the infused ones. Clinical and experimental studies of the anesthesia effect on ischemic preconditioning should be conducted in the future to determine its effect.

Although the exact mechanisms by which RIC reduces ischemic injury in the brain remain unclear, the currently accepted hypothesis is that preconditioning, perconditioning and postconditioning are all involve in both humoral and neural mechanisms^12^. RIC has been successfully reproduced by dozens of experimental laboratories but translation to the human clinical setting is still a challenge^6^. Despite many clinical trials shown protection to the heart, large randomized controlled trials found no improvement in clinical outcome and mortality in patients undergoing coronary bypass grafting^72^. Several trials are currently ongoing to explore the effects of RIC in ischemic stroke patients^73^. Data from these trials will help to better understand the effectiveness of RIC in AIS patients and will guide potential future implementation of RIC in the clinical practice.

The current systematic review and meta-analysis is the most recent revision of the literature on preclinical studies of RIC. It has considered three RIC strategies individually to define its effects independently, by contrast two recent systematic reviews and meta-analyses^74,75^ considered only two RIC strategies (RIPreC vs RIPerC + RIPostC; RIPreC vs RIPostC). We have considered a subgroup analysis by species (mice, rats, monkeys) because of the vascular hemodynamics of each specie. A detailed summary of the three systematic reviews is provided on Table 3.

**Table 3:** Comparison between two recent published metanalysis on RIC and our current metanalysis.

	Weir et al.*	Ripley et al.**	Torres-Querol et al.
**Methods**			
Data bases	Embase Medline Pubmed Web of science	Embase Medline	Pubmed Web of science SCOPUS
Searched up to	December 2019	August 2019	March 2020
Extracted data (outputs)	Infarct volume Neurological deficit	Infarct volume	Infarct volume Neurological function
Results expression	SMD	SMD	% infarct reduction
Nº publications	57	72	60 (more recent articles)
Groups of RIC	2 (pre and per + post)	2 (pre and post)	3 (pre, per and post)
Data analysis	Species Model of ischemia (permanent vs transient) Time of administration (Pre or Per + Post) Dose (number, length and total length of cycles) Study quality	Species Model of ischemia (permanent vs transient) Duration of ischemia Infarct volume assessment Time of administration (Pre or Post) Dose (type, number and length) Number of limbs Type of anesthesia Sex	Model of ischemia (permanent vs transient) Duration of ischemia Time of administration (Pre, Per or Post) Dose (number and length of cycles) Number of limbs Start of conditioning Type of anesthesia Sex
**Results/conclusions**			
Average reduction in infarct volume (all studies)	SMD − 1.87 ≅ 34% reduction	SMD − 2.19	− 38% reduction
Efficacy of RIC (all studies)	RIPerC/RIPostC > RIPreC in both rats and mice	RIPost > RIPerC	RIPerC and RIPostC > RIPreC in rats RIPreC > RIPerC and RIPostC in mice
RIC reduced lesion volume	Transient = permanent Length of RIC cycle > 5 min Total length ischemia > 10 min Number of cycles < 4 One = two limbs	Male > female No significant difference: Number of limbs Number of cycles and sessions Duration of O/R Species	Transient > permanent Length of RIC cycle > 15 min Number of cycles < 3 One = two limbs Male > female Type of anesthesia (with isoflurane less reduction)
Subgroup analysis	By type of administration (pre or per/post)	By sex, stroke type, type of RIC, conditioning, anesthetic	By species
Include the underlying mechanisms contributing to RIC	No	No	Yes

^a^Ref. ^75^.

^b^Ref. ^74^.

Some potential limitations should be stated. First, a large proportion of studies included in the meta-analysis use young male rodents with an absence of animals with co-morbidities which may inhibit the effects of RIC and a lack of adults/aged animals. In clinical studies, RIC would be used to treat aged persons with hypertension, diabetes and dyslipidaemia, which are not represented in preclinical models at this time. Second, considering that the incidence of stroke is higher among women compared to men, with women experiencing poorer outcomes, it is imperative to include more females in future studies. Third, anesthesia during RIC delivery is another concern because it is reportedly neuroprotective in preclinical models of stroke. Fourth, apart from infarct volume, we did not perform the meta-analysis of neurological function outcome because it was reported in a wide variety of different tests which make the analysis weak and deficient, and the high variability might be complicated to understand.

## Conclusion

This article, to our knowledge, is the first meta-analysis of RIC in preclinical stroke models that includes data on lesion volume, neurological impairment and mechanisms involved in RIC. This study demonstrated that RIC is a feasible and safe strategy and supported the ability of RIC to reduce infarct size and improve neurological function. However, the present study detected moderate statistical heterogeneity across studies influenced by species. Precise knowledge of RIC optimal dosage, the effects of comorbidities, sex and anesthesia is yet to be found. Further investigation in pre-clinical characterization of the RIC protocol obeying animal research guidelines is needed so that it can be successfully translated to humans.
